# Семейная форма нефрогенного Х-сцепленного несахарного диабета

**DOI:** 10.14341/probl13098

**Published:** 2022-07-13

**Authors:** В. В. Клепалова, О. С. Пушкарева, Н. В. Изюрова, А. В. Аксенов

**Affiliations:** Южно-Уральский государственный медицинский университет; Южно-Уральский государственный медицинский университет; Южно-Уральский государственный медицинский университет; Южно-Уральский государственный медицинский университет

**Keywords:** несахарный диабет, наследственный нефрогенный диабет, дети, полиурия, полидипсия, гены AQP2 и AVPR2

## Abstract

Отмечается мировая тенденция к росту распространенности несахарного диабета. Симптомы нефрогенного несахарного диабета (ННД) при Х-сцепленном наследовании проявляются у мужчин, у женщин с гетерозиготными мутациями, характеризуются изолированным симптомокомплексом полиурии, полидипсии, гипостенурии. У детей чаще, чем у взрослых, при ограничении жидкости развивается клиническая картина вододефицитной дегидратации с гипер­натриемией, гипертермией и гиперосмоляльностью плазмы. В данной рукописи представлен случай ННД, семейная форма, Х-сцепленная у пациентов мужского пола. При этом в семье по женской линии у матери и бабушки также наблюдалась повышенная потребность в воде, применение минирина было неэффективно. У старшего и младшего братьев клинические проявления несахарного диабета в виде выраженной жажды и полиурии были отмечены с младенческого возраста, после обследования выставлен диагноз несахарного диабета и назначен препарат десмопрессина. Вследствие отсутствия эффекта от применения десмопрессина был проведен методом полимеразной цепной реакции и последующего прямого секвенирования анализ экзонов и прилежащих участков интронов генов AQP2 и AVPR2. В гене AQP2 мутаций не выявлено. В гене AVPR2 выявлена гемизиготная замена S315I. Подтверждена семейная Х-сцепленная форма ННД. Рекомендован гипотиазид, на фоне постоянного приема которого отмечается лишь незначительная положительная динамика.

## Abstract (en)

There is a global trend towards an increase in the prevalence of diabetes insipidus. Symptoms of nephrogenic diabetes insipidus with X-linked inheritance appear in men, in women with heterozygous mutations, are characterized by an isolated symptom complex of polyuria, polydipsia, hypostenuria. In children, more often than in adults, with fluid restriction, a clinic of water-deficient dehydration develops with hypernatremia, hyperthermia, and plasma hyperosmolality. This manuscript presents a case of Nephrogenic diabetes insipidus, X-linked familial form in male patients.At the same time, in the family along the female line, the mother and grandmother also had an increased need for water, the use of minirin was ineffective. In the older brother and younger brother, clinical manifestations of diabetes insipidus in the form of severe thirst and polyuria were noted from infancy, after the examination, the diagnosis was made — diabetes insipidus and desmopressin was prescribed.Due to the lack of effect from the use of desmopressin, the analysis of exons and adjacent sections of the introns of the AQP2 and AVPR2 genes was carried out by PCR and subsequent direct sequencing. No mutations were found in the AQP2 gene. The hemizygous substitution S315I was found in the AVPR2 gene. The familial form X was confirmed — linked nephrogenic diabetes insipidus. A hypothiazide was recommended, against the background of constant intake of which only a slight positive trend is observed.

## АКТУАЛЬНОСТЬ

Несахарный диабет (НД) — это заболевание, при котором происходит выделение больших объемов мочи с низким удельным весом (полиурия). Распространенность несахарного диабета в популяции составляет 0,004–0,01% (US Census Bureau, Population Estimates, 2004). Считается, что НД одинаково часто страдают как женщины, так и мужчины. Пик заболеваемости приходится на возраст 20–40 лет [1][2]. Наследственный (врожденный) НД проявляется изолированным симптомокомплексом полиурии, полидипсии, гипостенурии или встречается в структуре наследственного заболевания как один из синдромов [3]. Нефрогенный несахарный диабет (ННД) проявляется как приобретенное заболевание у большинства пациентов, но очень редко имеет наследственное происхождение. В настоящее время известно 4 наследственных формы врожденного ННД у детей и взрослых пациентов: Х-сцепленный рецессивный, обусловленный мутациями гена АVРR2, аутосомно-доминантный иаутосомно-рецессивный, обусловленные мутациями гена AQP2, ННД вследствие мутаций генов SLC14А1 и SLC14А2, кодирующих транспортеры мочевины UT-1 и UT 2 [4–6]. Примерно в 90% случаев он обусловлен дефектами рецептора к вазопрессину (V2) и имеет Х-сцепленный тип наследования. В остальных случаях заболевание наследуется аутосомно-рецессивно (9%) или аутосомно-доминантно (1%) и обусловлено мутациями гена AQP2, кодирующего регулируемый вазопрессином водный канал собирательных трубочек почек. К настоящему времени описано 36 мутаций гена AQP2 [7][8]. ННД вызван неспособностью клеток почечных собирательных протоков реагировать на антидиуретическое действие аргинина вазопрессина. Действие этого антидиуретического гормона в почках регулируется тремя подтипами рецепторов AVP, связанных с G-белком. Клетки почечных собирательных протоков не могут реабсорбировать воду, и почки производят большое количество мочи с низкой концентрацией в результате мутаций гена AVPR2 у пациентов с ННД Х-сцепленного типа. Дети, страдающие ННД, эмоционально лабильны, раздражительны, у них отмечаются снижение памяти, невнимательность, рассеянность, неугомонность или заторможенность, астеноневротический синдром, задержка физического развития. Постоянные жажда и полиурия определяют поведенческий стереотип детей с врожденным ННД. У детей доминирует стремление к утолению жажды и мочеиспусканию. При ограничении пациентов с ННД в жидкости развивается клиническая картина вододефицитной дегидратации с гипернатриемией, гипертермией и гиперосмоляльностью плазмы [9].

## ОПИСАНИЕ СЛУЧАЯ

Нами представлен случай ННД, семейная форма, Х-сцепленная, у пациентов мужского пола, наблюдавшихся в эндокринологическом отделении МБУЗ ДГКБ №8 города Челябинска.

Впервые в эндокринологическое отделение для дообследования и лечения пациент А. был переведен из отделения реанимации и интенсивной терапии в возрасте 1 года 9 мес с жалобами на выраженную жажду, полиурию более 3 л в течение суток, периодические интенсивные головные боли.

По данным анамнеза жизни, мальчик рожден на сроке 39 нед с массой при рождении 3750 г, длиной 51 см. Физическое и нервно-психическое развитие в первые годы жизни соответствовало возрастным нормативам. Перенесенные заболевания — редко ОРВИ. Аллергоанамнез не отягощен. Травмы, оперативные вмешательства и переливание крови отрицает. При выяснении семейного анамнеза установлено, что в семье по женской линии у матери и бабушки ребенка отмечена повышенная потребность в воде (мама выпивала и выделяла с мочой до 8–10 л в сутки, бабушка — около 7–8 л в сутки), со слов мамы ребенка, назначение ей минирина было неэффективно, в связи с чем самостоятельно был отменен, другого лечения не было назначено.

## Анамнез заболевания

У пациента А. с младенческого возраста нарастали жажда и полиурия (со слов матери, мог выделять с мочой более 3 л в сутки, пропорционально выпитому объему жидкости). В возрасте 1 год 8 мес поступил в детское дошкольное учреждение, где ребенка ограничивали в приеме жидкости, вследствие чего у мальчика начали появляться и со временем нарастать отеки, нарушалось самочувствие, появлялось изменение сознания вплоть до сопора, в связи с чем был госпитализирован в отделение реанимации, где были проведены необходимые лечебные мероприятия с положительным эффектом. При проведении лабораторно-диагностического скрининга обращали на себя внимание следующие показатели: в анализах мочи — плотность мочи 1002, снижение осмолярности мочи (240 мОсм/кг), в биохимическом анализе крови — гипернатриемия свыше 148 ммоль/л. Учитывая жалобы на полидипсию, полиурию (3–5 л/сут) со сниженной плотностью мочи, семейную отягощенную наследственность и анамнез заболевания с характерными клиническими проявлениями, ребенку выставлен диагноз: Несахарный диабет центрального генеза. Назначено лечение десмопрессином в дозе 0,1 мг в сутки, примечательно, что в течение года доза была увеличена до 0,3 мг/сут.

Учитывая отсутствие необходимого наблюдения и обследования (отказ родителей), ребенок получал назначенную терапию бесконтрольно в течение 8 лет. В возрасте 10 лет мальчик был повторно обследован в эндокринологическом отделении, где было установлено, что на фоне регулярного приема десмопрессина 0,3 мг/сут нарастали симптомы полиурии и полидипсии до 7–10 л за сутки. Одновременно был обследован и второй ребенок, 4 лет, из данной семьи с клиническими проявлениями НД в виде выраженной жажды и полиурии (более 4–5 л в сутки), которые, со слов мамы, отмечались с возраста 3 мес, а в возрасте 7 мес ему был также выставлен диагноз: Несахарный диабет. Назначен препарат десмопрессина.

## Результаты физикального, лабораторного и инструментального исследований

При осмотре ребенка А. состояние средней степени тяжести. Самочувствие не нарушено. Мальчик гиперстенического телосложения. Кожа — бледно-розовая, умеренная сухость кожных покровов. Сыпных элементов не отмечено. Подкожножировой слой развит избыточно, распределен равномерно. Язык не увеличен, налета нет. Зев — небные дужки не гиперемированы. Над легкими ясный легочный перкуторный звук. Дыхание при аускультации везикулярное по всем полям, дополнительные шумы не выслушиваются, с частотой дыхательных движений 18 в минуту. Границы сердца соответствуют возрастным нормам. Тоны сердца — ритмичные, звучные, с частотой сердечных сокращений 80 в минуту. Живот — мягкий, безболезненный. Печень и селезенка не пальпируются. Диурез — полиурия, никтурия. Стул — регулярный. Щитовидная железа — не увеличена. Половые железы сформированы правильно по мужскому типу — G1 Р1, яички в мошонке. Таннер 1.

Вес — 58,500 кг, ИМТ — 26,3 кг/м2, SDS ИМТ — +2,78, рост — 149,5 см, SDS роста — +1,12, показатели соответствует ожирению 2-й степени.

При лабораторном обследовании: показатели общего анализа крови в пределах нормы. В общем анализе мочи обращало на себя внимание снижение относительной плотности мочи — 1001, остальные показатели были в пределах нормы. Анализ мочи по Нечипоренко: лейкоциты — 1750 в 1 мл, эритроциты — 750 в 1 мл. При проведении пробы по Зимницкому: дневной диурез составлял 3855,0 мл, ночной диурез — 3630,0 мл, сниженный удельный вес мочи (1000–1003) был выявлен во всех порциях собранной мочи. Основные электролиты: Nа+ — 146 ммоль/л, К+ — 4,0 ммоль/л, мочевина — 5,5 ммоль/л, креатинин — 76 мкмоль/л, остаточный азот — 2,55 ммоль/л, осмоляльность мочи — 100 мОсм/кг, осмолярность плазмы — 312 мОсм/кг. Пробу с сухоядением полностью провести не представилось возможным, так как через 2 ч у ребенка резко ухудшилось самочувствие, отмечалось повышение температуры до субфебрильных цифр. Через 2 ч от начала прекращения употребления жидкости отмечена осмоляльность мочи 133 мОсм/кг. При проведении пробы с десмопрессином относительная плотность мочи сохранялась пониженной — 1004. По результатам ультразвукового исследования почек и надпочечников выявлена двусторонняя пиелоэктазия (справа до 7 мм, слева до 9 мм). Умеренное снижение зубца Т при электрокардиографии.

На электроэнцефалографии выявлены легкие признаки внутричерепной гипертензии. МРТ гипофиза не показало изменений, при обследовании глазного дна, функции щитовидной железы, надпочечников изменений не отмечено.

Неврологом выставлен диагноз: Мигрень простая.

## Лечение

Учитывая отсутствие эффекта от ранее назначенного и регулярно принимаемого десмопрессина в дозе 0,3 мг в сутки, ребенок был направлен в ФГБОУ «НМИЦ эндокринологии» г. Москвы, где был проведен методом полимеразной цепной реакции и последующего прямого секвенирования анализ экзонов и прилежащих участков интронов генов AQP2 и AVPR2. В гене AQP2 мутаций не выявлено. В гене AVPR2 выявлена гемизиготная замена S315I. На основании данного заключения был выставлен диагноз: Нефрогенный несахарный диабет, семейная форма, Х-сцепленная, средней степени тяжести. Рекомендован гипотиазид в дозе 50 мг 3 раза в день, постоянно, аспаркам.

## Исход и результаты последующего наблюдения

На данный момент известно, что после 3 лет постоянного приема гипотиазида у ребенка значимой положительной динамики не отмечено: сохраняются полиурия, полидипсия (8–10 л в сутки). При проведении ультразвукового исследования почек отмечается умеренная отрицательная динамика (расширение лоханок до 8–10 мм), лабораторно: функция почек сохранена.

У младшего брата обсуждаемого пациента на основании прямого секвенирования также в гене AVPR2 была выявлена гемизиготная замена S315I, что подтвердило диагноз: Нефрогенный несахарный диабет, семейная форма, средней степени тяжести. Он принимал модуретик, затем гипотиазид, но также сохраняются проявления НД (диурез–7–8 л в сутки).

## ОБСУЖДЕНИЕ

Врожденный ННД — редкое наследственное нарушение, характеризующееся отсутствием антидиуретического эффекта при приеме вазопрессина, проявляющееся повышенным выделением низкой плотности мочи и выраженной жаждой. Тяжесть заболевания варьируется от легкой формы с полиурией и полидипсией до тяжелого обезвоживающего криза с анорексией, снижением физических показателей, лихорадкой и запором [1][2]. В нашем случае у пациента с возрастом нарастает избыточная масса тела вплоть до ожирения. Женщины в большинстве случаев являются здоровыми или бессимптомными носителями. В нашем случае у мальчика были эпизоды обезвоживания, в том числе из-за ограничения потребляемой жидкости. В связи с полиурией и полидипсией появляется бессонница, чему сопутствуют физическая и психическая астенизация [9]. При Х-сцепленном наследовании у большинства пациентов отмечаются психические и эмоциональные нарушения — головные боли, бессонница, эмоциональная неуравновешенность вплоть до психозов, снижение умственной активности. Представленные пациенты имеют нормальный интеллект, при этом страдают мигренеподобной болью.

Мутации гена AVPR2, связанные с X-сцепленным ННД, были впервые обнаружены Ван ден Оувеландом в 1992 г. На данный момент известно в общей сложности 254 различные мутации генов AVPR2 [7]. Генетический анализ наших пациентов показал аналогичные мутации, и они были унаследованы от матери и бабушки, которые имели легкие проявления заболевания. Знание действия рецептора, вызванного мутацией в гене AVPR2 или носителе, поможет обеспечить адекватный уход и лечение пациентов с врожденным НД. Целью лечения пациентов с НД является обеспечение надлежащего баланса жидкости и предотвращение умственной отсталости из-за сильного обезвоживания, особенно в неонатальном периоде и раннем младенчестве. Основное лечение — обеспечение адекватного и свободного потребления жидкости, приверженность низкосолевой и низкобелковой диете, назначение нестероидных противовоспалительных средств, тиазидных или калий-сберегающих диуретиков по необходимости либо индометацина. Препараты гидрохлортиазида в настоящее время — единственные назначаемые препараты у пациентов с семейной формой ННД, но они не всегда имеют положительный эффект. Требуются дальнейшее изучение и разработка препаратов с выраженной доказанной эффективностью у данных пациентов.

## ЗАКЛЮЧЕНИЕ

НД — относительно редкое заболевание, которое является проблемой не только медицинской, но и социальной, и психологической. При появлении жажды у ребенка необходимо своевременно диагностировать заболевания, сопровождающиеся выраженной полидипсией, так как ограничение жидкости в детском возрасте часто является причиной тяжелых метаболических нарушений. Своевременная правильная диагностика данного заболевания позволяет избежать тяжелых осложнений, назначать адекватное лечение. Основным методом лечения НД центрального генеза до настоящего времени остается назначение десмопрессина, в то время как подтвержденный ННД требует назначения гипотиазида или сочетания гипотиазида с индометацином или с амилоридом. Учитывая, что несахарный Х-сцепленный диабет в настоящий момент является заболеванием с неблагоприятным исходом, детям с клиническими проявлениями и лабораторными показателями ННД рекомендовано проведение медико-генетического обследования.

## ДОПОЛНИТЕЛЬНАЯ ИНФОРМАЦИЯ

Источник финансирования. Поисково-аналитическая работа и подготовка статьи проведены на личные средства авторского коллектива.

Конфликт интересов. Авторы подтверждают отсутствие явных и потенциальных конфликтов интересов, связанных с публикацией настоящей статьи.

Участие авторов. Все авторы внесли значимый вклад в подготовку рукописи, прочли и одобрили финальный вариант.

Согласие пациента. Законным представителем пациента подписано информированное согласие на публикацию персональной медицинской информации в журнале «Проблемы эндокринологии» в обезличенной форме.

Благодарности. Выражаем благодарность за проведенное молекулярно-генетическое исследование отделению наследственных эндокринопатий ФГБУ «Национальный медицинский исследовательский центр эндокринологии» Минздрава России.
